# Parental experiences of using continuous glucose monitoring in their young children with early-stage type 1 diabetes: a qualitative interview study

**DOI:** 10.3389/fcdhc.2024.1479948

**Published:** 2024-11-14

**Authors:** Alison G. Roberts, Alexandra S. Tully, Sabrina K. Binkowski, Keely R. Bebbington, Megan A. S. Penno, Amanda J. Anderson, Maria E. Craig, Peter G. Colman, Tony Huynh, Kelly J. McGorm, Georgia Soldatos, Peter J. Vuillermin, John M. Wentworth, Elizabeth A. Davis, Jennifer J. Couper, Aveni Haynes

**Affiliations:** ^1^ Department of Endocrinology and Diabetes, Perth Children’s Hospital, Perth, WA, Australia; ^2^ Children’s Diabetes Centre, Telethon Kids Institute, The University of Western Australia, Perth, WA, Australia; ^3^ The University of Adelaide, Robinson Research Institute, Adelaide Medical School, University of Adelaide, Adelaide, SA, Australia; ^4^ School of Clinical Medicine, Faculty of Medicine, University of New South Wales, Sydney, NSW, Australia; ^5^ Department of Diabetes & Endocrinology, The Children’s Hospital at Westmead, Sydney, NSW, Australia; ^6^ Melbourne Health Pathology, Department of Diabetes and Endocrinology, The Royal Melbourne Hospital, Parkville, VIC, Australia; ^7^ Department of Endocrinology and Diabetes, Queensland Children’s Hospital, South Brisbane, QLD, Australia; ^8^ Children’s Health Research Centre, Faculty of Medicine, The University of Queensland, South Brisbane, QLD, Australia; ^9^ Department of Chemical Pathology, Mater Health Services, South Brisbane, QLD, Australia; ^10^ Monash Centre for Health Research and Implementation, School of Public Health and Preventive Medicine, Monash University, Melbourne and Diabetes and Vascular Medicine Unit, Monash Health, Melbourne, VIC, Australia; ^11^ Faculty of School of Medicine, Deakin University and Child Health Research Unit, Barwon Health, Geelong, VIC, Australia; ^12^ Walter and Eliza Hall Institute of Medical Research, Melbourne, VIC, Australia; ^13^ Department of Diabetes & Endocrinology, Women’s and Children’s Hospital, Adelaide, SA, Australia; ^14^ The University of Western Australia (UWA) Medical School, Pediatrics, Perth, WA, Australia

**Keywords:** type 1 diabetes, children, continuous glucose monitoring, early-stage type 1 diabetes, staging, monitoring, islet autoimmunity, parents’ perception

## Abstract

**Aim:**

To explore parents’ experiences of using continuous glucose monitoring (CGM) in their young children with early-stage type 1 diabetes, being followed in the Australian Environmental Determinants of Islet Autoimmunity (ENDIA) study.

**Methods:**

Parents of children with persistent islet autoimmunity who enrolled in the ENDIA CGM sub-study were invited to participate in an optional interview. Semi-structured phone interviews were conducted by a single researcher using an interview guide developed by a multi-disciplinary team. Interviews were conducted following a single CGM monitoring period and prior to parents receiving feedback on their child’s glycemic status. Following transcription, thematic analysis was conducted to determine common themes.

**Results:**

Nine parents (8 mothers, 1 father) were interviewed corresponding to ten children, with a mean (SD) age of 5.6 (2.2) years, who wore CGM for 97 (0.1)% of the time during their monitoring period. Three main themes were identified: (1) Information empowers and helps to reduce uncertainty; (2) Families’ acceptance of using CGM; and (3) Involvement in research provides support and preparation for the unknown.

**Conclusions:**

Parents reported a *p*ositive experience of their young child wearing blinded CGM, and the children tolerated wearing CGM very well. Parents were empowered by knowing they would receive information on their child’s glucose levels and patterns and felt well supported. This study provides novel insights into parents’ experiences of using CGM in very young children with early-stage type 1 diabetes.

## 1 Introduction

Over the past decades there have been significant advances in the understanding of the natural history of type 1 diabetes (T1D). Studies have shown that the peak-age for developing islet autoantibodies, indicating the initiation of the autoimmune process underlying T1D, is 9-30 months of age (1). Children with persistent multiple islet autoantibodies (≥2 antibodies detected in serial blood tests) have a 75% 10-year risk, and 100% life-time risk of developing clinical T1D (1). The resulting paradigm shift is that individuals with persistent multiple islet autoantibodies with normal glucose levels are now considered as having stage 1 T1D (2, 3). Those with islet autoantibodies and abnormal glucose levels without symptoms of diabetes are considered as having Stage 2 T1D, while Stage 3 T1D refers to those who meet the biochemical criteria for T1D either without (Stage 3a) or with symptoms (Stage 3b) (2, 3).

Impaired glucose homeostasis, as measured by fasting glucose, HbA1c and oral glucose tolerance tests, is known to start months, to several years, before the symptoms of T1D (4, 5). More recently, the use of continuous glucose monitoring (CGM) in individuals identified as having Stage 1 and 2 T1D has provided additional metrics of dysglycemia occurring prior to clinical presentation and diagnosis (6, 7) including glycemic variability measured as Standard Deviation of sensor glucose levels, Coefficient of variance and percent CGM time spent above various thresholds (8). Current CGM studies have predominantly been conducted in children aged >6 years (4, 6, 7) with limited data available for young preschool aged children, who may represent a more rapidly progressive phenotype of T1D (1, 9).

The Environmental Determinants of Islet Autoimmunity (ENDIA) study, an Australia-wide pregnancy-childhood cohort study, following 1,473 infants with a first degree-relative diagnosed with T1D, provides a unique opportunity to investigate glycemic progression in early-stage T1D. (www.endia.org.au) (10). The ENDIA study includes comprehensive longitudinal data and biological sample collection 3-monthly from birth to 2 years of age, and 6-monthly thereafter to age 10 years. Antibody testing for insulin (IA), glutamic acid decarboxylase 65 (GAD), tyrosine phosphatase-like insulinoma antigen (IA2) and zinc transporter 8 (ZnT8) is conducted at each study time point (9), to identify development of persistent islet autoimmunity (defined as islet autoantibody detection on ≥2 more occasions at least 3 months apart).

Since 2021, ENDIA study children with persistent islet autoimmunity have been invited to participate in the ENDIA CGM sub-study (ACTRN12620000947909) when persistent islet autoantibodies (defined as ≥2 islet autoantibodies to either IAA, IA2-A, GADA, ZnT8 detected in consecutive venous blood samples taken at least three months apart) are detected. Children participating in this sub-study undergo blinded Dexcom G6 CGM monitoring for a minimum of 14-days, every 3 to 6 months (11). The Dexcom G6 CGM system consists of a sensor, transmitter and receiver; the sensor which is ~3 x 4.6 cm in size, is inserted just under the skin on either the upper abdomen or upper buttock via a disposable applicator, continuously measures interstitial glucose levels every 5 minutes for up to 10 days (https://www.dexcom.com/en-us/g6-cgm-system). The transmitter wirelessly sends the glucose values via Bluetooth to the receiver where they are stored. For this study, blinded CGM was used following consultation with the ENDIA study consumer reference group, to minimize parental reaction and anxiety associated with real-time glucose readings. A maximum of three sensors were worn to enable a minimum of 14-days CGM data to be collected (11).

As studies on parental experiences of using CGM in young children with early-stage T1D are lacking, the aim of this small qualitative study was to increase understanding of the impact of using blinded CGM in this population.

## 2 Materials and methods

### 2.1 Study design

A qualitative descriptive approach was used which recognizes the subjective nature of the topic, and inherent differences in individual experiences (13). This approach enables data to be collected on the lived experience and perceptions of individuals, which in this study was the parents of young children with pre-symptomatic T1D, wearing blinded CGM prior to clinical diagnosis of T1D.

### 2.2 Study population

Parents of children with persistent multiple islet autoimmunity enrolled in the ENDIA CGM sub-study, were invited to participate in an optional interview to share their experience of CGM monitoring in their child. Parents were approached by their ENDIA research nurse sequentially once their child enrolled in the CGM sub-study. As the purpose of the interviews was to understand parental experiences related to their child’s wearing of blinded CGMs, interviews were scheduled following completion of CGM monitoring and prior to parents receiving feedback on the CGM findings.

An iterative analysis approach was used whereby the data from each interview was reviewed as it was collected (13). After completion of seven interviews, two independent study research personnel determined that no new data was being identified. The research team met and agreed that all Australian States should be represented in the study sample, therefore two additional interviews were conducted. A total of nine interviews were completed and analyzed.

### 2.3 Sample size considerations

To ensure a sufficient sample size for this small study was met, the concept of information power was considered, i.e. the larger information power the sample holds the lower the number of participants are needed (12). For this study, the specific study aim, purposive sampling method and detailed description given by parents meant that the data obtained was sufficient for the study aim of describing parental experiences of their young child wearing CGM.

### 2.4 Parent interviews

The study team comprised of pediatric endocrinologists, qualitative researchers, research nurses conducting the ENDIA CGM sub-study visits and a clinical psychologist developed an interview guide (**Table 1**). Semi-structured phone interviews were then conducted between February 2021 and November 2021. All interviews were conducted by the same researcher (AR), and participant consent was obtained to allow them to be recorded. AR is a research nurse in the ENDIA study and ENDIA CGM sub-study, with an established track record in conducting qualitative research. She was the ENDIA study nurse for one of the parents interviewed.

**Table 1:** Interview guide.

1) How did you feel about using the sensor/CGM on your child? 2) How did your child and other family members feel about using the sensor/CGM? 3) Did you/your child encounter any difficulties while wearing the sensor/CGM? 4) Did you do any finger pricks during the period of sensor/CGM use? 5) Have you made any changes to your diet or other aspects of your lifestyle since your child started wearing the sensor/CGM? 6) What was your motivation for participating in this CGM study? 7) What was your expectation for participating in this CGM study? 8) Did you choose to receive feedback from the CGM study period? 9) What will you do with this feedback? 10) If no: Why did you choose not to receive results from CGM? 11) What have you told your child about why they are wearing the sensor/CGM?

### 2.5 Data analysis

At the completion of each interview, the recording was deidentified, transcribed and the transcription validated for accuracy and completeness. Three researchers, (AT, SB and AR) read and reread transcripts independently to become familiar with the data prior to inputting it into the NVivo 12 software management package (14).

Using an inductive thematic approach, as outlined by Braun and Clark’s six-phase framework (15), each of these researchers independently read and reread the data, sorting it into initial codes. Researchers then met on a regular basis to consolidate codes and identify common themes. All members of the research team were included in the final phase of analysis. This included reviewing and consolidating codes and themes identified by AR, SB and AT to reach group consensus on the final themes and ensure credibility. The consolidated criteria for reporting qualitative research (COREQ) were followed (16), and an audit journal maintained at each data analysis step described above, documenting analysis decisions made and ensuring a transparent and repeatable approach.

## 3 Results

### 3.1 Participant characteristics

Nine interviews were completed in relation to 10 different children enrolled in the ENDIA CGM sub-study, as one parent was a mother with two children who completed separate interviews relating to each child’s experience of CGM monitoring (**Table 2**).

**Table 2:** Demographic and clinical characteristics of parents and their children.

PARENTS	
Number interviewed (n)	9
Mothers (n)	8*
Fathers (n)	1
Mean (SD) age at time of interview (years)	41.3 (5.2)
**CHILDREN**	
Number wearing CGM	10
Mean (SD) active CGM wear time (%)	97 (0.1)
Girls n (%)	6 (60)
Mean (SD) age at time of CGM (years)	5.6 (2.2)
Mean (SD) age at time of persistent islet autoimmunity detection (years)	2.3 (1.7)
Mean (SD) duration of persistent islet autoimmunity at time of CGM (years)	3.2 (1.8)

*one mother interviewed had 2 children in the ENDIA study.

Eight participants were mothers, and one was a father. The father and four of the eight mothers lived with T1D themselves, the remaining four mothers had either a partner or other child living with T1D (**Table 2**). The mean (SD) of parents at the time of their interview was 41.3 (5.2) years. At this time, the mean (SD) age of their children 5.6 (2.2) years and mean (SD) duration of persistent multiple islet autoimmunity 3.2 (1.8) years (**Table 2**). The children wore CGM for 97 (0.1)% of the time during their monitoring period. Reasons for reduced wear time included e.g. the sensor falling out accidentally or the receiver being >6 meters from the child for an extended period.

### 3.2 Interview findings

The mean (SD) duration of phone interviews was 33.5 (5.3) minutes, ranging from 29.6 to 41.5 minutes. Group consensus after thematic analysis of the interviews resulted in three main themes being identified (1): Information empowers and helps to reduce uncertainty (2); Families’ acceptance of using CGM; and (3) Involvement in research provides support and preparation for the unknown.

#### 3.2.1 Information empowers and helps to reduce uncertainty

Parents were aware that the risk of their child developing clinical T1D was increased due to their having persistent islet autoimmunity. Therefore, all parents were keen to obtain more information and understanding regarding their child’s glucose levels and patterns. Parents felt this information would improve their knowledge about their child’s risk of progression.

*‘Yeah, I know it’s not going to change anything and it’s not going to tell us if [child] will have diabetes next week, but it gives some kind of awareness and I guess educating for her as well…’* (Participant 07)

All parents expressed some apprehensions about receiving the results from their child’s CGM session and being hopeful it would not show any abnormality in glucose levels. However, despite this, parents felt that no matter what the CGM results showed, the knowledge would enable them to be more prepared, thereby, preventing possible complications such as diabetic ketoacidosis (DKA) or delays in starting insulin therapy.

*‘We can have that insight into … how is her body actually going at producing insulin, is it still doing its job, are we getting closer to a diagnosis or symptoms.’* (Participant 05)

‘*There is comfort knowing that the results are given to you … if it is going to happen, we need to deal with it, we can’t really just ignore it’ … ‘We don’t want to delay treatment.’* (Participant 03)

#### 3.2.2 Families’ acceptance of using CGM

##### 3.2.2.1 Parental experience

Parents described their overall experience and their perception of their child’s overall experience of CGM as being positive. The wearing of CGM by their child did not cause additional concern to them or their child, and most parents reported that blinded over non-blinded CGM was preferable, otherwise they may have felt concerned that they needed to respond to the sensor glucose levels.

*‘less distracting or concerning when it’s blinded.’* (Participant 04)

*‘I know, because she’s got antibodies, I worry. But I don’t extra worry when she’s wearing it [CGM], … I’m not fazed by it. I’m sure someone that hasn’t had to deal with diabetes and stuff before would probably feel a bit funny, but I’m used to it.’* (Participant 08)

Parents reported that all children were happy to wear the CGM, with some children proudly showing it off to their peers and taking ownership of the device.

*‘If he’s at school he likes showing it off, and he actually did a presentation at school when he had his first one put on, to show them and you know everything else at school. So, he was really excited by it.’* (Participant 02)

*‘[My child] was absolutely happy to wear her CGM monitor. She was really good we let her take charge of putting it on charge every night before bed it became part of her routine of going to bed.’* (Participant 01)

Parents reported that their child tolerated the sensor insertion and wearing of the CGM well. Parents mentioned that for some of the young children, the sensor tape was uncomfortable, causing skin irritation and in some cases the tape removal was more upsetting than the sensor insertion.

*‘Sometimes she says it gets a little bit itchy and hurts, but yeah.’* (Participant 08)

*‘He does pick at the sticker that’s around it you know that sort of irritates him a little bit. But in regard to having, it on, he is actually really good.’* (Participant 01)

##### 3.2.2.2 Other family members’ experience

Other family members were accepting of the child wearing CGM. Most families in the ENDIA CGM sub-study had previous knowledge or experience of CGM. Most siblings of the child wearing CGM, associated the CGM as something fun and felt jealous that they were missing out.

*‘So, her brother was jealous, he wanted to have that cool little bag and a sticker on his belly. But obviously everyone else in our family, we kind of know it’s, I guess it’s normal in our world to wear a sensor.’* (Participant 07)

*‘…they were just quite happy to go for it, kind of thing. Obviously, my mother-in-law and father-in-law have a better understanding because they dealt with my husband when he was diagnosed and …, they were just amazed about how far technology has come. But no, everyone was really supportive so…’* (Participant 05)

#### 3.2.3 Involvement in research provides support and preparation for the unknown

All parents commented that they valued being part of the main ENDIA study as they belonged to a supportive group and felt happy that they were contributing to advancement of research into T1D. Additionally, it provided them with an opportunity to learn about, and access current research and intervention studies.

*‘I think ENDIA as a whole has been a great source of information for us, to be a bit more aware and a bit more conscious that they are more likely to have diabetes.’* (Participant 07)

*‘Um, just so there’s more research information to see if it helps in any way. So hopefully we can prevent or stop diabetes. The more information people get the better to try and fix it’* (Participant 08)

All parents mentioned that the staff rapport and trust they have developed over several years within the ENDIA study was an important contributor to their experience in the ENDIA CGM sub-study.

*‘I think our team managed it really well because everyone was really supportive, [Doctors name]. she was checking in with us and giving us a lot of information and the ability to ask questions and I think she was always on call when we needed her.’* (Participant 10)

*‘[Staff member] is a ball of knowledge as well, like you can ask her anything and she’s got an answer to it!… She knows how to speak to children as well as parents.’* (Participant 03)

## 4 Discussion

To our knowledge, this is the first qualitative study describing parental experiences of using CGM in their very young children with persistent islet autoimmunity, or early-stage T1D. CGM is now regarded as an essential component of optimal management of clinical T1D and is associated with improved glucose levels, improved sleep, and a greater sense of safety in parents using remote monitoring when away from their children (17). Although most studies in children with clinical T1D have reported positive health and quality of life outcomes when using CGM, barriers and anxiety about CGM have also been reported (18). As global islet autoantibody screening efforts expand (19), the use of CGM for glycemic monitoring and staging of T1D is under increasing consideration (6). CGM offers advantages but also some limitations in monitoring of pre-symptomatic T1D when compared to more traditional laboratory-based methods (20, 21).

This study reports findings from an inductive thematic analysis of semi-structured interviews exploring the parents’ perspective of their young child with persistent multiple islet autoimmunity wearing blinded CGM for a minimum of 14 days. All parents interviewed reported an overall positive experience. Specifically, parents reported no additional burden from their child wearing blinded CGM. Rather, the knowledge they anticipated to gain regarding their child’s glucose levels was expected to reduce uncertainty about their child’s progression to clinical onset, which was a perceived benefit from participating in the ENDIA CGM sub-study. This information was welcomed by parents irrespective of what the CGM findings might show about their child’s glycemic status.

An important finding reported by parents was their preference for their child wearing blinded rather than unblinded CGM, as they were not distracted/influenced by real-time glucose levels, and therefore felt less worried. This is consistent with feedback obtained from the ENDIA consumer and patient reference group prior to development of the ENDIA CGM sub-study protocol, whose overarching position was that blinded CGM was preferrable for minimizing burden and anxiety during the CGM session for families participating in the sub-study. Further rationale for using blinded CGM in the ENDIA CGM sub-study was to minimize participant-initiated changes to health behaviors (e.g., dietary intake) in response to real-time glucose readings.

This study provides novel insights into parents’ perspectives and experience of using CGM in young children with pre-symptomatic T1D and reported benefits gained from having more information on their child’s glycemic status and disease progression. Importantly, these findings may not be generalizable to parents in families without a first degree relative diagnosed with T1D or those not actively involved in research. Participants in this study were self-selected and may be more motivated to engage with CGM monitoring and be more accepting of the technology than those in the general population. Future research is needed to understand the experiences and perspectives of families without a family history or prior knowledge of T1D, to determine the acceptability of using CGM in this population. Further, the perspective and experience of the children wearing CGM themselves needs to be determined. Although not included in the aims of this study due to the very young age of the ENDIA CGM sub-study children, parental perspectives in this population provides relevant key insights as the connection between caregivers and young children appears to play a pivotal role in creating a favorable experience of research, as we have previously reported (22). Future research is also needed to determine the acceptability of longitudinal CGM monitoring, from the perspective of both parents and children, using appropriate validated measures to explore psychosocial impacts and include interviews at multiple time points.

Notwithstanding these limitations in the interpretation, the reported findings on the lived experience of parents and families on using CGM in young children with pre-symptomatic T1D provide novel insights from parents that are highly relevant to informing the development of acceptable glycemic monitoring approaches for families with very young children at risk of clinical T1D (3).

## Data Availability

Deidentified participant data will be made available to investigators whose proposed use of the data has been approved by an independent review committee (“learned intermediary”) identified for this purpose. Requests for data can be made by email to the ENDIA Study Chief Operating Officer at endia@adelaide.edu.au.
